# Development of next-generation antimicrobial hydrogel dressing to combat burn wound infection

**DOI:** 10.1042/BSR20203404

**Published:** 2021-02-08

**Authors:** Zlatko Kopecki

**Affiliations:** Future Industries Institute, University of South Australia, Adelaide, Australia

**Keywords:** antimicrobial hydrogel, biofilm, burn wound infection

## Abstract

Infection of burn wounds often leads to poor healing, sepsis, disability, or even death. Traditional care focuses on early debridement, fluid resuscitation, and intravenous antibiotics but these are often inadequate due to compromised vasculature limiting systemic antibiotics effectiveness. Biofilms in burn wounds are barriers to treatment and are associated with the transition of wounds from acute to chronic non-healing state. Current topical treatments for burn wounds include skin substitutes impregnated with skin or stem cells that promote healing; or hydrogels delivering an antibiotic, silver, or synthetic antimicrobial peptides. The success of currently available products is varied and, in some cases, very limited due to associated cytotoxicity to mammalian cells, the ability to only fight extracellular biofilm infections, and the ever-increasing development of antimicrobial resistance (AMR). There is, therefore, a high clinical need for the development of next-generation hydrogel wound dressings, to combat bacterial burn wound infection. A recent paper by Khan et al. (*Bioscience Reports* (2020) **39**, https://doi.org/10.1042/BSR20190504) highlights the development of a catechol cross-linked antimicrobial peptide hydrogel, adding to the body of literature describing innovative solutions with better delivery systems for antimicrobial peptides, and identifying a promising future biomaterial for development of novel hydrogel dressing to combat multi-drug resistant bacterial infections in burn wounds.

Wound infection and sepsis are serious complications for burn victims as significant thermal injuries induce a state of immunosuppression that predisposes them to a life-threatening condition. A high density of microorganisms on the burn wound or graft bed can lead to biofilm development. Indeed, the majority of burn wounds are colonized by bacterial biofilms and this significantly disrupts the healing process resulting in impaired functional and structural wound tissue. Invasive infection is also common when the presence of microorganisms is found adjacent in unburned tissue. Management of infected burn wounds involves debridement followed by dressings and antimicrobials to suppress biofilm reformation [1]. However, despite optimal topical antimicrobial therapy, antibiotic stewardship, and early and aggressive debridement and grafting, burn wound infection; including sepsis, cellulitis, and graft loss; still occurs in over 20% of burn patients [2]. While the survival rates for burn patients have improved substantially over the last two decades, the systemic inflammatory response syndrome, sepsis, and multiple organ dysfunction syndrome remain major causes of morbidity and mortality [2].

An additional problem in the management of burn wound injuries is an increasing rise in antimicrobial resistance (AMR) which is becoming a severe threat for burn patients. Bacterial pathogens use virulence factors to enable biofilm formation and are difficult to treat as they possess highly developed strategies for eluding eradication including multidrug efflux pumps, antibiotic-modifying enzymes, and tough outer membranes with low permeability [3]. In fact, biofilms are known to prevent drug penetration into the infected burn wounds resulting in severe wound complications. Preventing or reducing wound infection would be a major leap forward in the clinical management of burn injuries. Current burn wound management is a complex process relying heavily on wound dressings which serve primarily to cover the wound and prevent infection. Current techniques to prevent burn infection include non-toxic wound cleansing, debridement of necrotic tissue, antibiotic management, and the use of moisture-retentive dressings [4]. Recent advanced technologies for burn wound management include the development of sustained-release silver or cadexomer iodine antimicrobial dressings, negative-pressure wound therapy, and dressings that incorporate hydrogels impregnated with biologics or agents that can stimulate burn repair or fight infection (e.g. silver or zinc oxide nanoparticles) [5]. However, despite the recent research progress, the success of these treatments remains clinically inadequate, as potential toxicity, stability, and lack of desirable antimicrobial release highlight some of the major treatment limitations. Besides, none of the current advanced dressings can provide multifunctional properties including the ability to stimulate healing, and simultaneously prevent infection, while reducing scarring in a burn injury. As an alternative, the advanced development of hydrogel-based dressings has gained considerable attention as the most promising drug delivery system. This has led to significant optimization and development of advanced hydrogel dressings to address various aspects of burn wound management including wound healing, scarring, and infection.

Advanced wound treatment is often based on the controlled delivery of active substances into the burn site. Hydrogels are widely available in the market as the most ideal wound dressings with numerous advantages for burn wounds including the ability to provide a moist and cooling environment beneficial for burn wounds, non-adhesiveness to wounds, and ability to absorb excess wound exudates. Additionally, the high-water content of hydrogels mimics the physiological wound conditions, favoring tissue regeneration with excellent biocompatibility, and the capability to encapsulate a variety of antimicrobial drugs. Additionally, it forms a protective barrier from pathogens and provides a physiological environment that could stimulate the natural self-healing mechanisms for fast healing. Besides, a hydrogel can be designed to trigger antimicrobial release in response to environmental stimuli. Stimuli-responsive moieties allow hydrogels on-demand volume transitions in response to the external environment including changes in pH, temperature, light, hence empowering widespread biomedical applications in wound dressings, artificial skin, and drug delivery [6]. Among all, a pH-responsive hydrogel is heavily exploited for improved delivery of antimicrobial to the wound site, where the release pattern can be tailored based on the pH of the wound site thereby providing specificity. As a result, hydrogel dressings are widely recognized as an important drug carrier for next-generation material for improved sustainability and therapeutic efficacy. Importantly, hydrogel fabrication is best recognized for the ability to fine-tune their physical, chemical, and biological properties depending on the purpose of treatment modality [5]. For example, the physical and chemical properties of hydrogels can be tailored to provide a desirable slow and sustained release of drugs for long-term application against bacterial biofilms with reduced toxicity to mammalian cells. In hydrogels, the extent of macromolecular cross-linking allows for tuning of the physical, chemical, and biological properties, which in the case of natural polymers is also coupled to the intrinsic biocompatibility [5].

While many natural hydrogels have inherent antimicrobial properties (e.g. chitosan, β-chitin, cellulose, dextran), many have been loaded with synthetic antibiotics and antibacterial agents including metal-ion loaded hydrogels, metallic-nanoparticle, AMP based-hydrogels, and natural polymer-based hydrogels bearing synthetic antimicrobials (for detailed review, see [7]). The progress in technology and synthesis in the last decade has expanded the array of novel antimicrobials and stimuli-responsive drug delivery hydrogels available to combat wound infection including thermogels, photocontrolled-release hydrogels, magnetic gels, and multiresponsive hydrogels (for detailed review, see [8]). Novel design strategies also include incorporation of sensor molecules into the hydrogel platforms to allow monitoring of the wound healing status as well as healing progress [5].

Clinically, hydrogels are used as wound debridement agents, moist dressings, and components of wound treatments. In burn wound management, hydrogels act as a moisture donor and can accelerate wound healing through autolytic debridement and moisture regulation [9]. The development of improved animal models of acute and biofilm wound infection in the last few years has facilitated increased testing of novel antimicrobials and stimuli-responsive drug delivery hydrogels [10–12], suggesting that more products can be expected to transition to the clinical setting in the coming years. The desirable properties of an ideal antimicrobial hydrogel wound dressings for the treatment of burn injury are presented in Figure 1.

**Figure 1:**
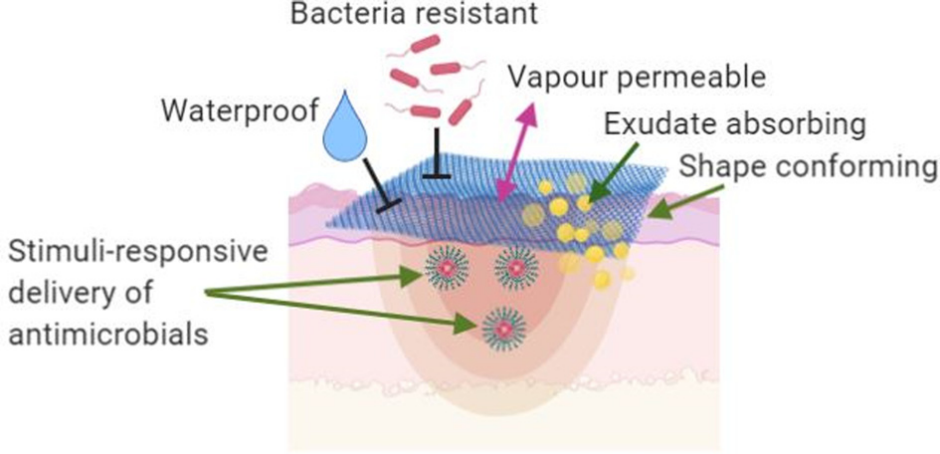
Desirable properties of the advanced antimicrobial hydrogel wound dressings for the treatment of burn wound injury

Current clinical trials are mostly focused on the post-market analysis of hydrogel dressings and clear results to date have shown that compared with standard of care hydrogels improve the appearance of the healed burn wounds, resulting in less painful dressing changes, improved wound re-epithelization, and reduced need for surgical excision or grafting [5]. However, no hydrogels to date have shown efficacy in both promoting burn wound regeneration and combating burn wound infection simultaneously. Additionally, the hydrophilic nature of hydrogels makes it difficult to displace water from adhesive interface, therefore, the development of more compatible, effective, and stable interfaces has been a strong research focus for the development of advanced hydrogel-based wound dressings.

A recent study by Khan et al. describes the development of a novel promising biomaterial to combat bacterial wound infection with good biocompatibility and no negative effects on burn wound healing [13]. The authors explore the unique chemistry of catechol featuring a combination of hydroxyl groups and phenol functional groups allowing bioadhesion to wet surfaces through a series of synergistic processes including *in-vivo* coacervation, surface spreading, phase inversion, and covalent cross-linking. The authors optimized the gel forming system by synthesizing hydrogels based on mole ration of epsilon-poly-l-lysine (EPL) to catechol (0.3:0.1; 0.4:0.1, and 0.5:0.1). EPL and catechol were first dissolved in Tris/HCl solution prior to 3-day incubation at 37 degrees to allow partial oxidation of catecholamine and followed by 2-day storage at 4 degrees to make the hydrogel. An increasing number of studies are using catechol organic compound as a substrate of choice in the development of functionalized dressings. Authors use this biomimetic hydrogel to deliver a known natural antimicrobial peptide EPL, which is composed of identical l-lysine residues characterized by the peptide bond of lysine monomers to γ-amino functional groups and carboxyl groups. The cross-linking scheme of EPL-catechol is illustrated in Figure 2A. Cationic antimicrobial peptides, like EPL, offer a promising opportunity to combat bacterial infections as they damage the bacterial cytoplasmic membrane and are less likely to trigger AMR development. Studies to date have shown that EPL has a broad-spectrum activity against Gram-negative and -positive bacteria and is biodegradable, non-toxic, and cost-effective for biomedical applications [14]. The novelty of this work lies in the cross-linking of catechol with ELP in a single-step process, refining the previously described complex chemical processes and eliminating the need for use of organic solvents. The authors characterize the porous structure of the hydrogel and anti-biofilm activity *in-vitro* and extend their studies to animal models.

**Figure 2:**
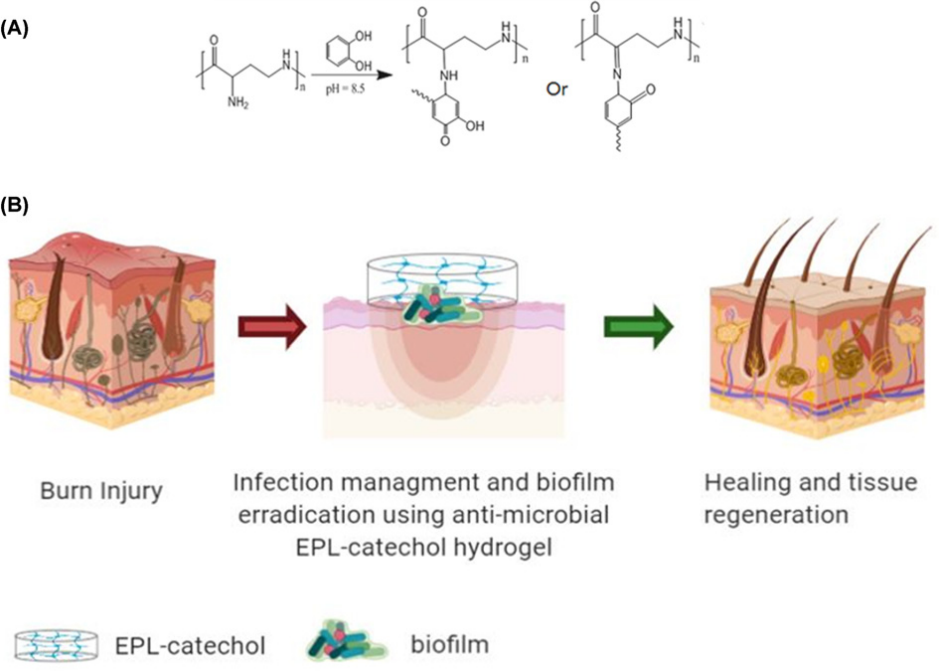
Next generation hydrogel system for treatment of burn wounds (**A**) EPL-catechol cross-linking scheme and (**B**) anti-biofilm efficacy towards the development of a novel hydrogel wound dressing for treatment of burn wound infection.

*Acinetobacter* infection is an increasing global problem for burn wound management as the incidence of this infection in patient population is increasing significantly, especially in developing countries. *Acinetobacter baumannii* infection is easily transmitted and viable for a long time in the hospital environment due to the multidrug resistance, resistance to desiccation, and tendency to adhere to inanimate surfaces. Using the partial-thickness burn injury model, followed by infection with a clinical strain of a multidrug-resistant Gram-negative *A. baumannii*, Khan et al., demonstrate the ability of the newly developed EPL-hydrogel to significantly reduce *in-vivo* biofilms in burn wounds (Figure 2) which is most likely attributed to the action of both EPL and function of reactive oxygen species (ROS) and hydrogen peroxide (H_2_O_2_) byproducts of catechol oxidation [13]. Indeed, this agrees with previous studies which have also described the use of injectable EPL nanocomposite hydrogel to combat infection and promote tissue healing and regeneration *in-vivo* [15]; however, exact mechanisms of actions in burn wounds are yet to be determined. The tunable functionalities of the EPL-catechol biomaterial make it a promising product for future investigation in biomedical applications. Lastly, the authors show a high degree of biocompatibility and cytotoxicity of the developed hydrogel using both cell lines and *in-vivo* tissue analysis with no signs of inflammation or toxicity upon topical or intradermal applications of the hydrogel, most likely attributed to the anti-inflammatory nature of catechol previously described [9].

While the described study presents advances in one-step preparation of EPL-catechol cross-linking and offers novel insights into EPL antimicrobial efficacy for treatment of burn wound injuries, future studies should investigate the efficacy of the developed hydrogel in bioluminescent models of burn wound polymicrobial infection which allow temporal monitoring of infection progression and more closely represent clinical burn wound environment. Moreover, further research is required to adequately understand the underlying effects of EPL-catechol on burn wound repair, including the exact mechanism of action and subsequent effects on inflammation signaling, collagen deposition, and angiogenesis. Previous research has shown that tuning the oxidation state of catechol using the changes in pH allows for repeat activation (high pH) and deactivation (low pH) of this biomaterial for on-demand generation of H_2_O_2_ [16]. Consequently, more research is required to explore if the developed hydrogel can be further functionalized for on-demand activity and to fully understand the effect of EPL-catechol curing chemistry, byproduct generation, and degradation products on the healing of infected burn wounds. Lastly, preclinical efficacy and safety studies in porcine animal models of burn wound infection need to be undertaken before this research can progress towards testing of EPL-catechol hydrogels in human clinical trials. Taken together study by Khan et al., suggests that the developed biomimetic EPL-catechol hydrogel offers a promising platform for the development of the next-generation advanced hydrogel wound dressing to combat infection in burn injuries [13].

## Abbreviations

AMR: antimicrobial resistance
EPL: epsilon-poly-l-lysine
H_2_O_2_: hydrogen peroxide
